# Ponds as experimental arenas for studying animal movement: current research and future prospects

**DOI:** 10.1186/s40462-023-00419-9

**Published:** 2023-10-25

**Authors:** Christer Brönmark, Gustav Hellström, Henrik Baktoft, Lars-Anders Hansson, Erin S. McCallum, P. Anders Nilsson, Christian Skov, Tomas Brodin, Kaj Hulthén

**Affiliations:** 1 Department of Biology—Aquatic Ecology, Lund University, Ecology building, Sölvegatan 37 223 62, Lund, Sweden; 2 Department of Wildlife, Fish and Environmental Studies, Swedish University of Agricultural Sciences (SLU), Umeå, 90183 Sweden; 3 National Institute of Aquatic Resources, Technical University of Denmark (DTU), Silkeborg, Denmark

**Keywords:** Lakes, Ponds, Movement ecology, Animal movement, Individual movement, Acoustic telemetry, Whole-pond telemetry studies, Experimental approaches, Hypothesis testing, Cause-and-effect relationships

## Abstract

Animal movement is a multifaceted process that occurs for multiple reasons with powerful consequences for food web and ecosystem dynamics. New paradigms and technical innovations have recently pervaded the field, providing increasingly powerful means to deliver fine-scale movement data, attracting renewed interest. Specifically in the aquatic environment, tracking with acoustic telemetry now provides integral spatiotemporal information to follow individual movements in the wild. Yet, this technology also holds great promise for experimental studies, enhancing our ability to truly establish cause-and-effect relationships. Here, we argue that ponds with well-defined borders (i.e. “islands in a sea of land”) are particularly well suited for this purpose. To support our argument, we also discuss recent experiences from studies conducted in an innovative experimental infrastructure, composed of replicated ponds equipped with modern aquatic telemetry systems that allow for unparalleled insights into the movement patterns of individual animals.

## Introduction

Most animals possess the ability to move, at least during a part of their life cycle, and, hence, movement is a fundamental feature of animal life. Animal movement is a multifaceted process that occurs at a broad range of spatial scales, spanning millimetres to continents, and animals move from one place to another for a variety of reasons [1, 2]. For example, animals may move to specific habitats to find food or mates and away from others to avoid predators, parasites, or harsh environmental conditions [3–5]. Consequently, understanding the underlying drivers of movement patterns has a pivotal role in our understanding of most ecological and evolutionary processes [1, 6]. If we are to fully understand the drivers behind different movement patterns among species and environments, we thus need to quantify key aspects of the movement repertoire in a reliable manner and with high spatiotemporal resolution.

Much of our knowledge regarding animal movement derives from observational field studies of relatively low precision, or from small-scale experiments in mesocosms or in the lab, all of which do not mirror the complexity or potential feedbacks occurring in more natural settings. However, the advent of new tracking methods, especially the development of small electronic transmitters, have revolutionized the study of larger-scale movement patterns directly in the wild. Specifically, technology now allows for collection of massive amounts of high resolution (in both time and space) data on movement behaviours relevant for interpretations of how individuals perceive and interact with the abiotic and biotic features of their environment [7]. Today, animal-borne equipment not only track the position of the animal, but may also provide data on its internal state, including physiology and energetics [8, 9]. By tapping into these approaches, we can now thoroughly test ideas and concepts born out of relatively low-resolution studies. Such approaches will not only advance our basic understanding of ecological and evolutionary processes, but also have the potential to revolutionize more applied disciplines like conservation biology and invasion ecology [10, 11].

Compared to the progress made in studying the behaviour of terrestrial animals in the wild, research on behaviour in aquatic ecosystems has been comparatively less developed. This has likely been a consequence of the challenges associated with acquiring high resolution, fine-scale behavioural data in aquatic environments. However, over the past decade, we have witnessed significant advancements in acoustic telemetry technology. In particular, the miniaturization of transmitters and the development of longer-lasting batteries, as well as improved communication protocols and techniques to transfer large amounts of data under water and enhanced data storage and analytical capabilities. These breakthroughs have enabled the collection and transfer of large amounts of data on free-roaming aquatic animals, providing researchers with previously unattainable levels of spatial and temporal resolution, precision, and accuracy [7, 12, 13]. Acoustic telemetry systems consist of transmitters that are attached externally or internally to study animals and that transmit ID-coded ultrasonic signals that are recorded automatically by receivers [14]. Multilateration of signals from receivers deployed in arrays with overlapping detection ranges then allows for simultaneous, high-precision tracking of the movement behaviour of a large number of individuals (e.g. [15]). Thus, we now have at our disposal a tremendous toolkit that can be used to study movement patterns, such as movement speeds, distances, trajectories, turning angles, and, importantly, their connection to ecological processes, including e.g. habitat use, interactions with competitors and predators, and effects of environmental factors.

The key question now is where and how to perform such studies. Recently it was suggested that lakes are ideal study systems for addressing the most fundamental and pressing questions of today in movement ecology [16]. Lakes are advantageous to work in because they are relatively closed systems with well-defined borders, allowing movement patterns to be linked to key environmental factors, including the abiotic factors that reign in the specific lake, but also to the composition of the biotic community that affect the strength of biotic interactions [17]. Studies performed in multiple lakes also allow for comparisons of how different environmental factors affect movement patterns (e.g. [18]). Clearly, there are several advantages associated with lake studies, although most animal movement studies on a lake scale have typically been restricted to observations of free-ranging animals in natural systems often focusing on “where and when”— i.e. researchers have been identifying and describing spatial and temporal movements patterns, such as diel and seasonal changes in space use, home-ranges, migratory patterns, and spawning sites of threatened species. But, to truly address the most significant evolutionary and ecological questions related to animal movement, we need to understand the underlying mechanisms and identify causality by controlling for confounding factors. To achieve this, we require replicated studies in controllable experimental environments where the effect of variables of interest can be isolated and tested against a baseline control.

While laboratory and mesocosm experiments can offer such controlled conditions, they often constrain experimental animals into confined and unnatural conditions, which may compromise their natural behaviour and potentially affect the validity of the results in reflecting processes in the natural world. To fill the gap between laboratory/mesocosm studies and whole-lake studies, we need replicated systems at a relevant scale where we have strong control over the species, populations, individuals and traits in the experiment, and where we can achieve complete coverage of the whole system, including tagging the entire fish assemblage. This is challenging in lakes but manageable in smaller pond systems, as proposed by Lennox et al. [16].

## Experiments need ponds

We suggest that ponds are particularly useful arenas for tracking studies, mainly because their fish communities can be fully controlled and manipulated, and, yet, ponds are large enough for key behavioural repertories to be expressed. Thus, ponds have an untapped potential that can pave the way for significant knowledge breakthroughs in movement ecology, given that extrapolations to larger scale systems are carefully weighed. Ponds are very common globally; in fact they are the numerically most dominant size category of standing freshwater systems [19], and thus provide an ideal experimental system for replicated study designs. Ponds often hold diverse and functionally complex (including multiple species of both predators and prey) fish communities that are relatively stable over time [20]. Even if small compared to many lakes, ponds are still of a large enough size to allow for key behavioural repertoires to be expressed, allowing us to use ponds for animal movement studies that are ecologically relevant. It is also important to note that ponds may exhibit substantial differences in community structure as well as in the abiotic environment and there may, hence, be large among-pond variation in putative ecological drivers expected to exert force on fish movement ecology that can be explored. There are a few studies that have implemented tracking with acoustic telemetry in ponds [21–23], but a common limitation is the lack of truly replicated experimental setups, which restrict the studies to experimental designs with consecutive temporal trials to achieve replication. To the best of our knowledge, no study has so far utilized the full potential of experiments in highly controllable and replicated pond environments and, therefore, we decided to develop an experimental pond infrastructure using a system of man-made ponds with similar physical characteristics. This setup, with multiple similar aquatic environments as experimental units, each equipped with modern tracking technology, opens up for robust designs, through inclusion of appropriate controls and replication, key aspects for inference, yet rarely included in movement ecology studies.

### The experimental pond infrastructure

**Fig. 1:**
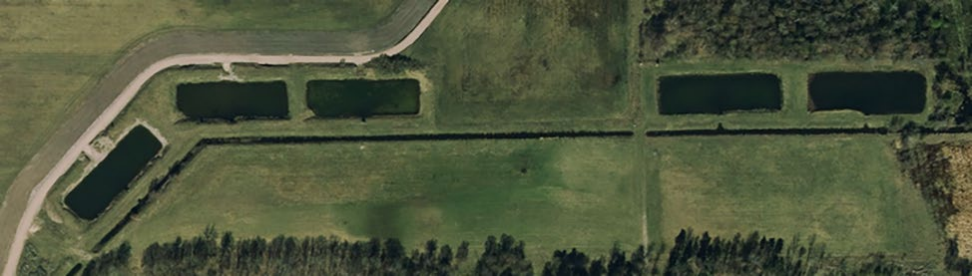
Aerial view of the pond facility showing replicate ponds. Running in parallel to the ponds is the small stream feeding all ponds with water via an adjustable inlet

**Fig. 2:**
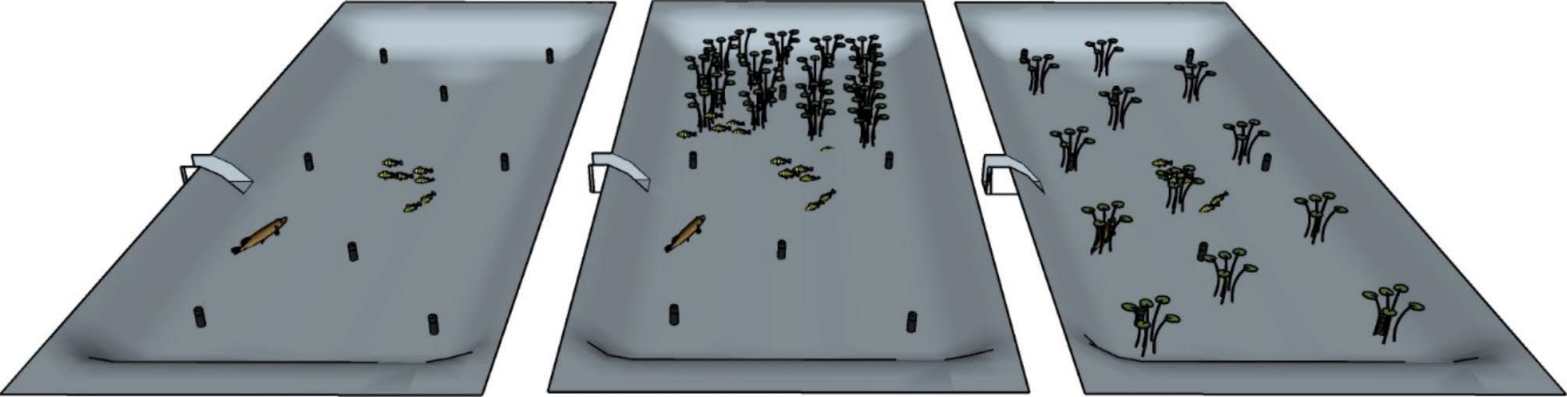
Schematic illustration of a manipulative experiment designed to investigate the impact of habitat complexity/patchiness on predator-prey dynamics/interactions. The predator is here represented by pike (*Esox lucius)*, and the prey by Eurasian perch (*Perca fluviatilis*). The black cylinders depict acoustic receivers configured in an array that enables fine scale positioning of tagged fish. In the left pond, predator and prey interact in an open habitat lacking complex habitats or refuges. In the central pond, aquatic plants are used to provide a refuge only in one end of the pond to create a heterogeneous risk environment. In the right pond, aquatic plants are distributed in patches, creating a heterogenous environment for predator and prey to navigate in. The iPonds allow for quantification of high resolution behavioural and physical parameters in both predators and prey, such as distance to predator, predation events, prey foraging and heart rates, which can then be related to habitat selection and prey decisions about distance-to-refuge and visual obstruction

The experimental pond infrastructure (a.k.a. iPonds) has been developed in collaboration with South Sweden Water Supply AB (Sydvatten AB), one of Sweden’s largest drinking water producers, and is located at the Vomb waterworks facility about thirty kilometres east of Lund, Sweden. The infrastructure hosts a series of dedicated ponds (Fig. 1; man-made infiltration ponds) of similar size and depth (~ 90 × 30 m, 1.5 m deep). The ponds have a sandy substrate, large areas of the ponds are generally covered with dense stands of submerged vegetation (mainly *Chara* spp.) and they are fringed with reed beds (*Phragmites australis* cav). In addition to representing morphologically similar habitats, all ponds also share the same water source as they are fed with water from nearby lake Häljasjön (55° 40′N, 13° 33′E) via a small stream running parallel to the ponds (Fig. 1). Each pond has an adjustable inlet (but no outlet) that provides us with the ability to regulate the water level and if completely closed we can almost entirely drain the pond. This feature is instrumental in granting us complete control over the fish assemblage as we can easily remove all fish individuals residing in the ponds prior to experiments and then re-stock fish to establish experimental populations, where each and every individual is tagged and where the fish community is assembled based on the exact requirements of the specific experiment (e.g. species, density, size structure). Moreover, we can easily enter the ponds at low water levels to manipulate habitat features, e.g. by removing vegetation patches or installing artificial, standardized refuges and/or cages with predators to manipulate perceived predation risk (see Figs. 1 and 2 for potential experimental designs), i.e. we can construct experimental “pondscapes” to explicitly test theories on, for example, resource availability-predation risk trade-offs. Finally, the ability to lower water levels enables us to efficiently recapture experimental fish for quantifying post-experimental changes in phenotypic traits (morphology, growth rate, immune defence, etc.) and, further, to retrieve fish with bio-logging devices.

**Fig. 3:**
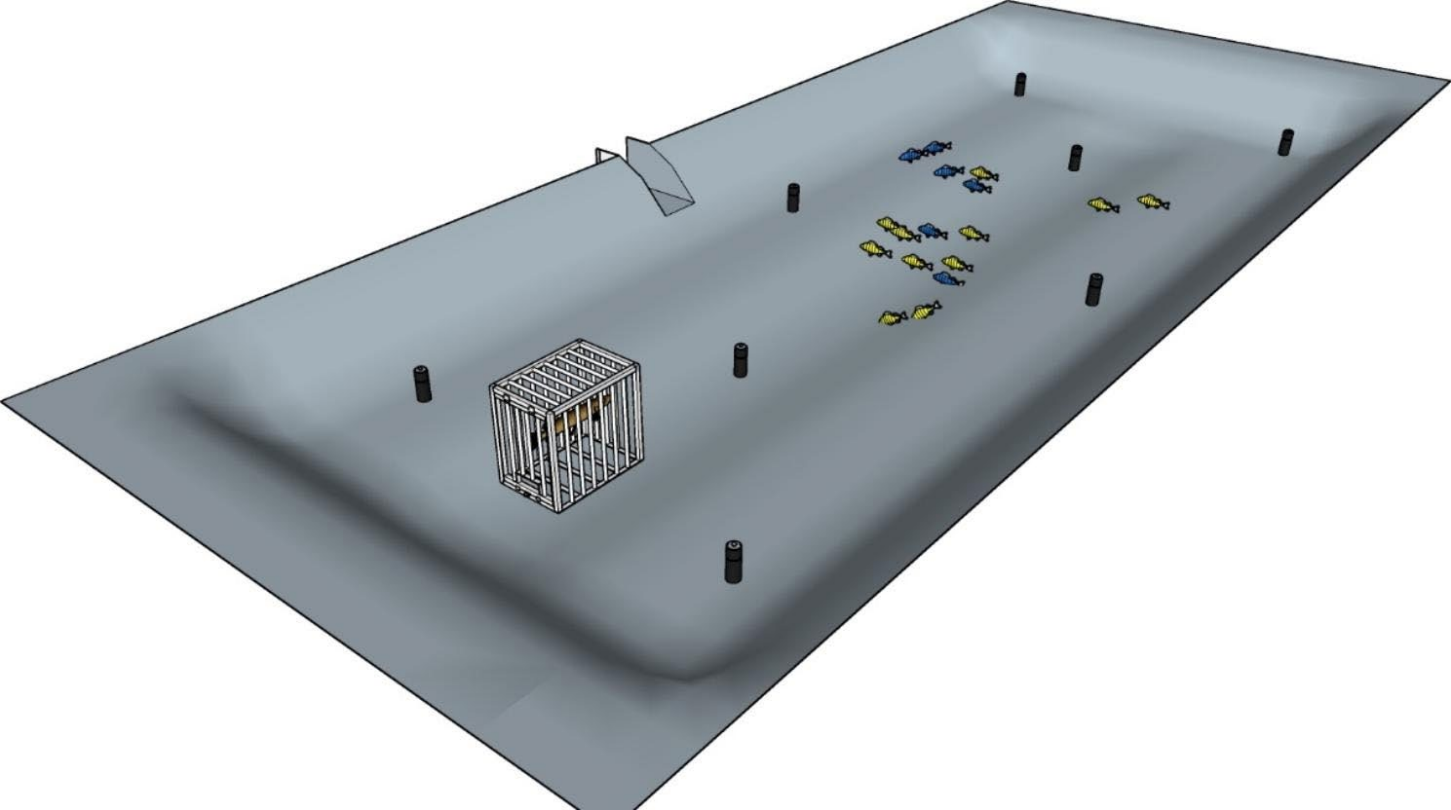
Schematic illustration of a manipulative experiment designed to investigate environmental pollution by pharmaceuticals on fish behaviour. Fish individuals are fitted with slow-release implants either containing (yellow) or not containing a contaminant (blue). Following controlled manipulations of pharmaceutical exposure, fish behaviour can be monitored using whole-pond high-resolution acoustic telemetry under different relevant ecological scenarios, including no predators, caged predators (as shown here) or freely roaming predators)

The ponds are equipped with modern acoustic telemetry equipment providing us with an unprecedented opportunity to autonomously collect high-resolution data on movement behaviours in completely known fish assemblages for extended periods of time. At its core, the technology consists of two principal components: ID-coded transmitting tags (surgically implanted into the body cavity of the fish) and data-logging hydrophones that are used to detect and store timestamped registrations of transmitter IDs [14, 24, 25]; several manufacturers offer transmitter/logger systems that allow for simultaneous tracking of hundreds of tagged fish in each pond (see [26] for further details on capacities and limitations of high-resolution tracking systems). Thanks to a dense receiver array, comprising eight submerged acoustic telemetry receivers anchored to the pond bottom at fixed locations (as shown in Figs. 2 and 3), transmitter signals are simultaneously detected by multiple receivers. This enables spatial positioning of fish-borne transmitters via multilateration and, hence, we can reconstruct the whole-pond movement paths of individual fish. The temporal resolution of the data partly depends on hardware configuration, such as the burst interval of transmitting tags, and can be pre-set according to experimental design and study-specific tracking needs. Because of the trade-off between battery life and sampling intensity, one may choose to configure the hardware to generate detections of fish individuals every second, suitable for example when quantifying inter-individual distances in studies of coordinated behaviour, or at longer time intervals to extend battery-life, in studies of long-time movement patterns, such as seasonal or even inter-annual habitat-related variations in movement ecology. Moreover, combining acoustic tags with different types of implantable bio-loggers, such as heart-rate tags, may allow us to overlay acoustic telemetry*-*derived indices of movement behaviour with continuous records of stress physiology. In addition, the ponds can easily be sub-divided with fine-mesh nets for experiments on a smaller scale but with higher replication and the ponds can also be readily fitted with e.g. mazes, underwater-cameras and altered habitats, making the system very flexible to meet a broad range of experimental requirements.

Fine-scale positional data with sub-meter resolution, as described above, is distinctly different from spatially discrete data which is commonly generated when using acoustic telemetry to track fish over larger areas. Spatially discrete data consists of time-stamped detections from single receivers within an array, and the spatial resolution could hence be several hundred meters. Such course resolution is often not sufficient to study detailed behavioural interactions, habitat preferences or even survival. Technologies with shorter detection ranges can be applied, such as RFID-telemetry, to achieve spatially distinct detections in ponds but such methods often result in significant temporal detection gaps without data on the fish as it is residing outside the range of the RFID-antennas.

### Experimental designs in the pond system

Replicated pond systems, such as the iPonds facility, allows for different approaches to experimental design, including (1) observational evaluation of individual behaviours in space and time, (2) sequential addition and/or removal of experimental treatments with comparisons to untreated controls (BACI-design), and (3) factorial designs with simultaneous data collection of differently manipulated subsets of individual fish (see Figs. 2 and 3 for examples of potential experimental designs). First, purely observational studies can be used for deepened and detailed knowledge on e.g. diel activity patterns and differences in movement behaviour among species and sizes of fish. Further, by quantifying traits of each individual fish prior to the start of the experiment, such as body length, weight, condition and morphology, we can link individual movement patterns to specific phenotypic traits. The ability to recapture fish at the end of the experiment allows us to relate how movement patterns affect changes in key traits (e.g. morphology) during the experimental period. Besides morphological traits, we can also perform laboratory-based behavioural assays prior to release in ponds, i.e. quantification of standing variation in a broad spectrum of behaviours (animal personality/behavioural syndromes). Fitting individuals assayed for behaviour with acoustic tags and releasing them into the experimental ponds can unravel links between behavioural traits, movement patterns and fitness outcomes (e.g. [27]). Further, such pre-experimental phenotyping may be combined with studies of social interactions, collective cognition and behavioural culture in groups of fish.

Second, a more experimental approach involves sequential changes in the abiotic or biotic environment using a BACI-design where control and treatment sites (ponds in this case) are simultaneously sampled before and after the experimental manipulation in impact sites [28]. Further, we can change habitat features by removing or adding structures that may act as predator refuges (Fig. 2), change food resource levels or even manipulate the abiotic environment, e.g. by manipulating turbidity, light levels or temperature to further our understanding of how changes in the biotic or abiotic environment affects individual behavioural performance.

**Fig. 4:**
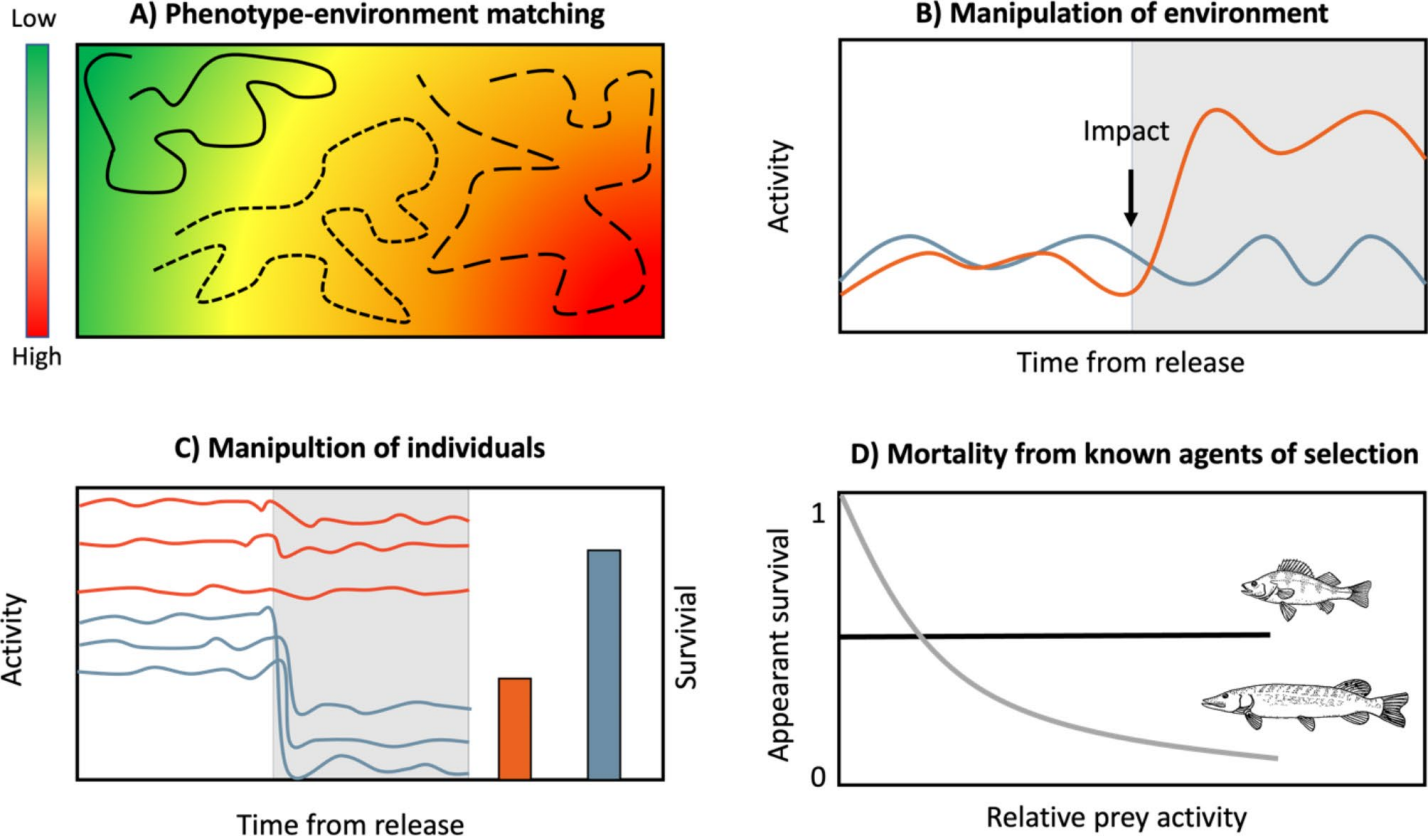
Examples of experimental scenarios in which *ponds* equipped with high-resolution *acoustic* telemetry system can serve as particularly useful empirical substrates **(A)** By releasing previously phenotyped fishing ponds with experimentally induced spatial variation in risk (e.g. caged predators) researchers can study to what extent individual prey phenotypes (behaviour, morphology, physiology) predict movement patterns and habitat choice of prey in a “landscape of fear”. **(B)** Whole-pond acoustic telemetry in combination with controlled environmental manipulations (e.g. water turbidity) allow for powerful comparison of continuous high-resolution individual-level data acquired pre/post-environmental-impact. **(C)** In ponds, researchers can also manipulate stocked individuals (e.g. via slow-release implants for manipulating contaminant exposures) and compare their movement patterns in relation to unmanipulated control individuals. By simultaneous tracking of such individuals through a change in ecological context (e.g. no predators (white section) versus caged predators (shaded section)) researchers can also unravel how the expression of key movement behaviours may depend on interactions between manipulations and ecological contexts. Finally, by the release of caged predators, the predation vulnerability of manipulated and control individuals can be directly assessed, to link behavioural effects to fitness (bars, right y-axis). **(D)** Fish-removal followed by re-stocking in ponds have several associated benefits and can for example allow researchers to immerse fish into a controlled predation-risk environment and link survival selection to an identifiable agent. Hence, one can expose fish to different kinds of predators (e.g. sit-and-wait predators (e.g. pike) versus active pursue foragers (e.g. perch) and ask if different types of predators generate similar or different patterns of correlations between key movement traits and viability

Lastly, the iPond infrastructure allows for factorial experiments where the movement of fish from different treatments is quantified simultaneously (Fig. 4B). For instance, by comparing the behaviour of individuals that have been experimentally manipulated prior to release into the ponds, for example by varying levels of perceived predation risk and/or food availability or manipulating their key sensory systems (e.g. olfactory acuity), with the behavioural performance of control individuals we can directly test mechanistic hypothesis on the importance of threat perception on individual behaviour.

## Emerging future directions

Recently, [29] listed a number of key questions in the study of movement ecology of marine megafauna, some of which could preferably be studied with acoustic telemetry methods, and this was further elaborated by [16], specifically for animals inhabiting lake systems. Here, we focus explicitly on the strengths associated with collecting high resolution movement data in pond settings and expand on emerging issues that could be explicitly studied in these environments. Further, experimental ponds may serve as a controlled, replicable environment where the functionality of acoustic telemetry systems under diverse environmental conditions could be investigated, including e.g. the influence of underwater noise on detection probability, the validation of sensors etc.

### Predator-prey interactions

Virtually all animals in nature are somehow engaged in predator-prey interactions and understanding this phenomenon constitutes a staple in ecology and evolution research [30–32]. In the past, a wide range of proxies have been used to estimate predator-mediated selection and impacts on prey populations, including metrics of spatiotemporal overlap between predators and their prey, predator diets inferred from gut content analyses and stable isotope techniques, direct observations of predation events and changes in prey behaviour and density after natural or manipulative changes in predator density (see e.g. [33]).

Obviously, changes in movement patterns in relation to prevailing predation pressure is a crucial component of predator-prey interactions, but mechanistic studies on this phenomenon directly in nature is extremely challenging. Hence, very few experimental studies have been able to disentangle the direct and indirect effects predators have on the movement of their prey on a temporal and spatial scale that is relevant for natural settings [16].

However, as outlined above, with the advent of high resolution, high throughput telemetry and its application in a replicated pond infrastructure we are now in a position where we with carefully designed experiments can dramatically increase our mechanistic understanding of the complexities of predator-prey interactions in movement ecology. A great advantage is that we have complete control of the fish assemblage in the experimental ponds and that we can tag and then simultaneously track all individuals, predators as well as their prey. This opens up for a range of studies on the movement ecology of predator-prey interactions, as exemplified below.

First, we focus on direct lethal predator-prey interactions. Acoustic tag data may allow for an accurate analysis of fine-scale movement patters at the different stages of the predator-prey cycle, i.e. search, encounter, attack, capture and digestion [33, 34]. For example, how will differences in prey density, movement speed, habitat use, and temporal activity patterns affect predator encounter probabilities in prey? Once a prey has been encountered and is within the predator’s reaction distance [35] will the predator attack and what factors determine attack rates in the field? How frequently will predators choose to not attack a prey even when it is within its reaction distance? What movement traits are affected by predator-induced selection (Fig. 4D) and do different predator species exert similar or different selection pressure on movement behaviours? Attack events could be determined from acoustic tag data, for example by sudden changes in speed, and use of accelerometers could yield even higher resolution data (e.g. [36, 37]). Further, attacks are not always successful, but success rates an be determined by analysing predator and prey movement after an attack. Coordinated movements between predator and prey and/or sudden loss of the prey signal can be used to identify a successful attack, but recent developments of specific predation detection tags could enable a more reliable identification of successful predation events [38]. After a successful capture, how long will it take predators to digest the captured prey and reach hunger levels that will trigger resumed foraging, and how is that dependent on prey and predator size ratios, temperature, and inter-individual variability in metabolic profiles? Such data could be compared to results from bioenergetics models and be used in predictions of predator effects in stocking, management, and conservation efforts. Further, from a prey perspective, how will activity patterns and habitat use be affected by temporal variation in satiation (hunger) levels in their major predators? Can prey detect and fine-tune movement responses such as activity levels and the distance maintained to predators in relation to their current feeding state?

Moreover, we can directly study the adaptive value of specific prey traits (e.g. size, body morphology, behaviour) for reducing predation vulnerability. For example, to what degree can diurnal variation in the intensity of key anti-predator behaviours, such as schooling intensity [39], be explained by diurnal variation in predator activity or predator foraging success, e.g. dusk and dawn in pike [40, 41]. In addition to between-species comparisons, we now know that there is also a considerable intraspecific variation in a number of prey traits that may be important determinants of susceptibility to predation, including size, sex, morphology, personality and parasite load [42–46]. By mapping the sex and phenotype of individual prey fish [27, 47] before they are introduced to the experimental ponds we can directly evaluate how intraspecific variation in ecologically relevant prey traits affect predator-prey interactions in the wild without suffering from the potential confounding effects associated with between-species comparisons [48, 49]. Conversely, we can unravel how disparate predator species which differ in their predominant hunting mode [50], such as ambush versus active searchers or solitary versus group foragers, influence movement strategies in their prey. Further, a large body of literature suggests that interactions between predators may have strong effects on predation rates and the stability of predator-prey population dynamics [51–53] and here we can directly assess how predator-predator interactions, including direct physical harassments, but also the risk and occurrence of kleptoparasitism and even cannibalism [54, 55] affect predator-prey interactions. By careful manipulation of the size structure and/or the species composition of the predator assemblage, we will be able to monitor how predator-predator interactions will affect home-range sizes and patterns of activity and spatial overlap, as well as interference (e.g. kleptoparasitism) and cannibalism.

### Landscape of fear

Predators obviously have a strong effect on prey populations through direct lethal effects (killing), but predators also affect the prey they do not kill through fear alone (a.k.a perceived predation risk) and these indirect, trait-mediated effects may actually be larger than the direct effects of predation [56–60]. Prey organisms commonly respond to changing levels of predation threat with changes in behavioural traits, with the goal to reduce predator encounter rate, for example by reduced general activity, schooling, changes in diel activity patterns, and/or habitat (refuge) use to decrease predator-prey space overlap. Thus, even when the direct rate of predation is low, fear effects can still have far-reaching impacts on a suite of prey traits related to movement, by influencing prey navigation and spatio-temporal distribution in the so called “*Landscape of Fear*” [61, 62]. One factor that affects the architecture of the landscape of fear is habitat complexity, where complex habitats may increase refuge availability and at the same time decrease piscivore foraging efficiency [63]. By removing parts of the submerged macrophyte stands in the experimental ponds, or by adding artificial structures, we can thus manipulate the physical landscape and, hence, refuge availability, and subsequently assess how such manipulations translate into changes in the perceived landscape of fear.

### The predator avoidance-foraging trade-off

**Fig. 5:**
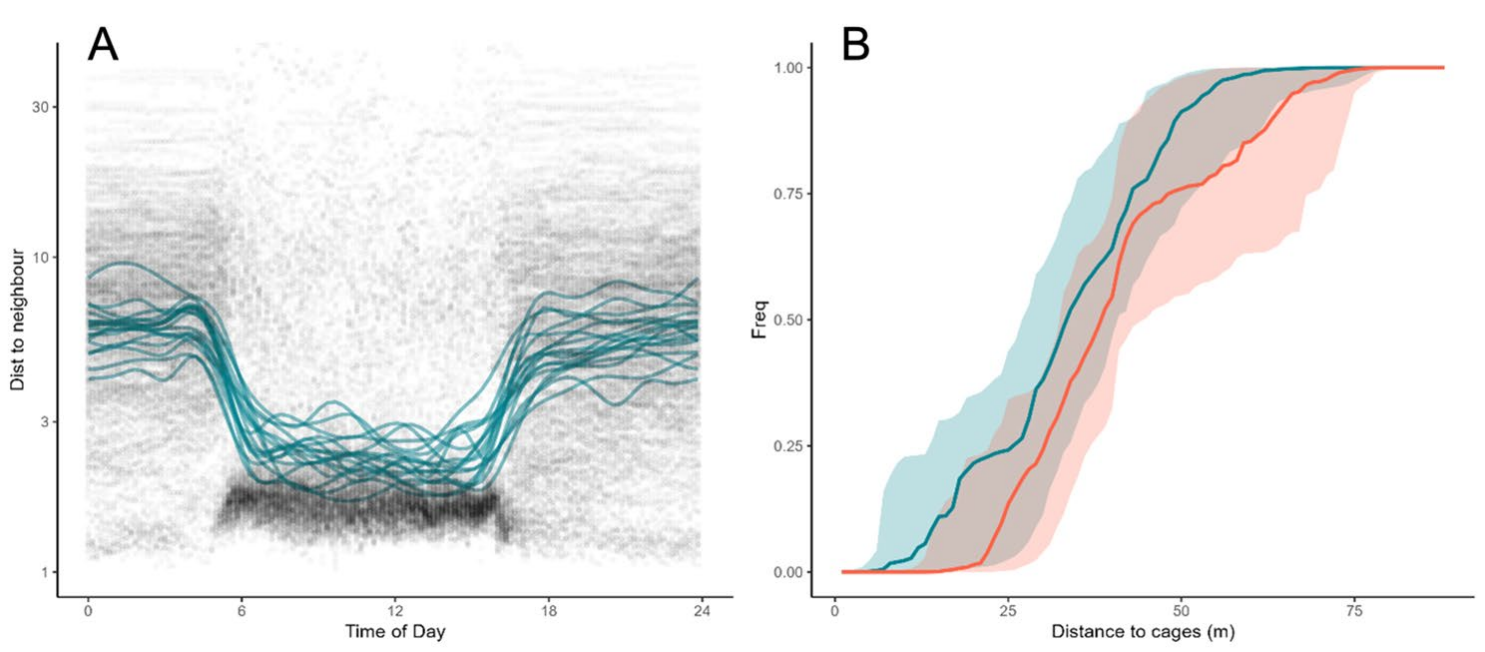
Examples of experimental data obtained using the iPonds system allowing for analysis of for example social interactions **(A)** and movement in a landscape of fear **(B)** **A**: Individual distances to nearest three conspecific neighbours were estimated for n = 17 tagged roach (*Rutilus rutilus*) individuals. Data shown are median distances in ten-minute intervals for a 14-day period (black dots) and an individual generalized additive model (lines). Diurnal dynamics of social interactions *(*distance to their nearest neighbour*)* are clearly visible and characterized by smaller inter-individual distances during day as compared to night-time **B**: Roach distances to four net cages during periods of seven days when the cage either contained (red) or not contained a pike (*Esox lucius*). For each day, individual empirical cumulative distribution functions (ecdfs) where estimated for each individual roach. Period specific 50% interquartile of these day-individual ecdfs were obtained and plotted. Lines represent median ecdfs

Although behavioural trait changes such as reduced activity and exploration can effectively reduce risk of predator-induced mortality in the short term, these changes naturally also incur costs, for example manifested as a reduction in time available for other activities, such as foraging [64]. When potential prey organisms navigate this landscape on a daily basis, they thus have to manage risk perception and perform critical risk-balancing trade-offs between, on the one hand, avoiding being preyed upon and, on the other hand, acquire enough recourses to fuel survival and reproduction. Furthermore, these trade-offs are by no means static because state changes in both prey and predators over time act to create a highly dynamic landscape of fear through time [65, 66] that prey organisms are forced to consider. For example, hunger levels in prey affect the willingness to take risks [67], and, further, cyclical changes in predator activity [65] on a seasonal or daily scale should affect the cost/benefit trade-off of avoiding predation in the landscape of fear. Simultaneously quantifying how foraging opportunities and risk of predation affect movement patterns in a dynamic landscape of fear seems like an unsurmountable challenge, but we suggest that experimental manipulations in ponds may offer a way forward (see Fig. 5B for preliminary data on behavioural responses to a caged predator). A recent study in a terrestrial system where voles where exposed to varying levels of risk and reward in an experimental landscape demonstrated that the Giving-Up-Density (GUD) of resources in a patch provides an efficient method to map risk avoidance-foraging trade-offs in a landscape of fear [68]. GUD is the food density at which the forager decides to leave the food patch as the benefits of foraging no longer outweighs costs and, hence, in a landscape of fear GUD reflects the perceived costs of avoiding predation in a foraging prey organism [69]. Quantification of GUD in a food availability context has been successfully performed in benthivorous fish by placing artificial patches with food pellets directly in lakes [70] and this method could easily be applied also in our experimental ponds in combination with manipulations of perceived predation risk, e.g. by placement of piscivorous fish in net cages (Fig. 3) and quantification of movement patterns of individual foragers. Similar studies on zooplanktivorous fish may be logistically more challenging but we envision that net cages that are stocked with zooplankton cultures where individual plankters can escape through the net can be used, thus creating experimental variation in food availability for zooplanktivorous fish. Again, experiments where zooplankton food patches are moved around in a landscape of fear as provided by piscivorous fish in cages and where zooplanktivorous fish movements are monitored should allow for a quantification of landscape of fear also for zooplanktivorous fish. Earlier studies have shown the feasibility of creating planktonic food patches and evaluating resource/threat trade-offs, in this case involving phytoplankton food and zooplankton grazers exposed to threats [71]. Hence, iPonds offers a possibility to quantify the perceived trade-off between opportunity and threat in disparate species of fish, thereby connecting our understanding through the food chain from zooplankton to fish.

### Individual differences in fear perception

While previous studies have focused on how *different species* perceive and respond to risk in the landscape of fear, a new exciting approach aims to shed light on how individual variation in traits *within species* influences cost-benefit trade-offs and perception of predator-induced fear. For example, in laboratory studies it has been shown that individuals from the same population differ in the degree to which they express plastic defence traits when exposed to chemical predator cues [72]. Will these inter-individual differences in anti-predator traits predict home ranges and how individuals move in the landscape of fear, i.e. are movement patterns based on phenotype-habitat matching (Fig. 4A)? An aspect of individual variation that has received particular attention is consistent individual differences in a broad spectrum of behaviours (sometimes referred to as “animal personality”), such as individual tendency to react to fear and take risks (boldness), exploration and sociality, but also in general activity levels [73–75]. The questions of what causes and maintains such differences in individual behaviour is fundamental, and here it has been suggested that predation risk is balancing the evolutionary maintenance and the costs and benefits of different personality types [42, 76, 77]. Individuals with risk-prone personalities may access greater rewards (e.g. food and mates), but at the cost of exposure to higher predation risk [78, 79]. This should contribute to non-random spatial sorting of phenotypes, i.e. prey adaptively choose to occupy space within the landscape of fear that best fits their personality or behavioural type [80]. However, direct evidence for variation in these cost-benefit trade-offs for free-ranging animals expressing different personality types in the landscape of fear are few (but see [42, 68, 78]). The importance of non-random distribution and specific movement strategies of disparate behavioural types in heterogeneous landscapes of fear could preferably be studied in experimental ponds using an experimental design in which we first assay individual behaviours of experimental fish in a controlled laboratory setting, acoustically tag them and then release them into the experimental ponds. A sequential change in predator context (no predators/caged predators/free-roaming predators) would allow for an evaluation whether individual differences in behaviour are consistent across lab- and field situations and also provide an explicit test of the degree of spatial sorting of phenotypes under low- and high-risk predation scenarios, as well as an assessment of the relative contribution of plasticity and consistent behaviour to spatio-temporal segregation of phenotypes.

### Acquiring information on predation threat

The ability to adaptively navigate in the landscape of fear requires that prey individuals can collect and accurately interpret information from the environment that reflects predator presence and the current risk it imposes [62]. In the aquatic environment, chemical cues (kairomones and alarm substances) is the most important way by which prey assess predation risk [81, 82], since auditory, visual and mechano-sensory mechanisms are often compromised. These cues can convey information regarding predator species, density, their specific diet and even hunger level. Hence, chemical cues do not only serve to reveal general predator presence, but also communicate information regarding the current risk posed by specific predators [83, 84]. A large number of studies in the laboratory or in small-scale field mesocosms have shown that chemical predator cues can affect behavioural responses of prey [81], but surprisingly few have taken the study of chemical predator cues into more realistic field settings and therefore we do not know how and at what scale prey organism react to chemical predator cues in nature [85–87]. Thus, the outstanding question is how do chemical predator cues affect individual prey perception of the landscape of fear in the wild? In a preliminary experiment in the iPonds facility we have monitored changes in prey fish movement patterns in response to changes in the perceived level of predation risk. During an initial phase we stocked an iPond with acoustically tagged prey fish and monitored their behaviour under a predator-free context. We then added a piscivorous fish (pike) to a net enclosure placed in the pond, i.e. prey fish were able to detect and evaluate predation risk by the presence of chemical predator cues (the piscivore where fed prey fish in the net cage). Another promising experimental approach to evaluate the importance of chemical cues for predator detection and navigation in a landscape of fear is to monitor movement patterns of a treatment group with an experimentally blocked olfactory sense compared to the movement of an untreated control group in which olfactory acuity remains intact.

### Physiology

Ample opportunities now exists to integrate whole-pond telemetry data with data on physiology and/or environment, for example the integration of positional data with high frequency bio-loggers via double-tagging approaches where acoustic tags are combined with e.g. acceleration or heart-rate sensors, or sensors for e.g. dissolved oxygen. This type of data fusion will provide a more holistic understanding of fish movement ecology in general and holds great promise in uncovering the interplay between the environment, fish movement behaviour and physiological state. With the use of high resolution bio-loggers, the experimental ponds such as the iPonds facility enable the causal chains between ecological interactions, physiological reactions and ultimately fitness to be studied under realistic conditions. The ability to recover tagged fish after the end of the study is key here, as it will allow for the collection of the very large datasets stored within the bio-loggers which cannot be transmitted under water with current technologies. Easy retrieval of all fish will also enable before-and-after measurements and biopsies of fish with regards to e.g. growth, stress-hormones and various metabolites. Continuous data on the physiological state of an individual can then be correlated to a detailed behavioural record containing all the social interactions, foraging attempts, habitat choices and predator attacks experienced by the same individual. In addition, combining data on movement patterns with analyses of gene expression may enable an evaluation of how selection pressures affect specific genes [88]. Here, the iPonds facility serves as a powerful new tool to address challenging multidisciplinary research questions previously restricted to confined laboratory experiments, for example how hormone levels or metabolic profiles affect behavioural responses to varying levels of predation risk or resource availability across time, or how the physiological state of individuals affect foraging efficiency, energy expenditure, decision-making processes or potentially even reproductive success under different environmental conditions [89–92].

### Environmental factors

Replicated ponds are an excellent venue in which to ask and answer research questions regarding the influence of external environmental factors on animal movement. This is, in part, because certain environmental factors can still be manipulated at the spatial scale of a pond that would not be feasible in larger lakes, riverine, or open-water habitats. First off, habitat structure and/or vegetation could be manipulated at the scale of replicated ponds to enhance, degrade, or change habitats by manipulating the type, amount, density, and/or clustering of habitats (Fig. 2). This type of experimental intervention allows researchers to both directly manipulate habitat for their research question(s) and also to control for habitat variation across ponds. Habitat manipulations could be used to ask questions about habitat choice, risk taking behaviour, and resource partitioning. Temperature is a timely environmental factor to manipulate to ask and answer questions related to the effects of habitat warming and climate change on animal movement. Temperature can be manipulated at larger scales via heat exchangers (see [93] for an example in smaller scale ponds). An interesting avenue for future research could be to manipulate water temperatures and water levels (for ponds with controlled water depth) to study the effects of drought scenarios on animal movement and habitat use.

Replicated ponds can also be used to study the effects of environmental contaminants on free-ranging fish movements at a natural scale. For example, fish could be either (a) exposed to a chemical contaminant in the lab and then released into the ponds [94], (b) exposed internally using slow-release or time-release implants [95], or (c) exposed directly in the ponds via whole-pond exposures [96, 97]. For options ‘a’ and ‘b’ to work, the metabolism and elimination rate of the contaminant from the fish would need to be known ahead of time to properly parameterize the study. Option ‘c’ would allow whole ecosystem type studies to be conducted where the response in more than just fish (e.g., aquatic invertebrate communities, trophic chains) could be measured. However, we argue that whole-pond exposures are not preferred as they can be very costly, and, more importantly, have lasting damages to the environment as contaminants that are not quickly metabolized in the environment are not easily eliminated from the water column and sediment.

In addition to environmental contaminants, excess nutrients or turbidity could also be manipulated in a replicated pond setup to study the effects of eutrophication or run-off from agriculture, forestry, or land-use change on animals movement. Likewise, light and noise pollution could be manipulated in ponds via the use of spotlights, underwater speakers or direct noise sources (e.g., boat motors, underwater drilling). It is important to note that manipulating certain environmental factors can have potentially long-lasting effects on pond habitats (e.g., long term contamination, altered benthic communities, disturbed water clarity). It is therefore important to carefully consider how extreme the manipulation is and how readily the pond set up will return to a reference state.

### Social interactions

Animals may live in groups for multiple reasons. Group living can reduce individual risks of predation via vigilance, confusion and dilution effects, benefit growth via social foraging, and enhance information processing and behavioural performance through collective cognition and animal culture in varyingly complex social networks [98–102]. Work in experimental ponds paves the way for precise and deepened understanding of such fish social interactions under natural conditions. For instance, high resolution individual positioning data can convey detailed school shape and cohesion information, and school positioning and leadership/follower characteristics of individuals within schools. As all fish identities can be known (equipped with tags) in experimental ponds, naïve individuals can be added to experienced fish groups to evaluate e.g. rate of information transfer, and fish can be left to regroup on a daily basis after switching to more solitary behaviours during night (see Fig. 4A for preliminary data in diurnal dynamics of social interactions) for analysis of assortative grouping by different phenotypes or individuals in social networks. Further, the emergence of school-specific behaviours creates links to the development of animal culture, and the high-resolution positioning data combined with fitness proxies such as individual growth opportunity and predation probability can shed light on how social interactions in general are exposed to and respond to ecological selection.

### Fish-angler interactions

Modern fishing techniques and equipment have significantly improved the efficiency of both commercial and recreational fishing, such as angling [103, 104], leading to a major transformation in the way that humans interact with fisheries resources. Hence, now is the time to explore new and innovative approaches to better inform conservation and management strategies for the sustainable use of exploited fish stocks.

In this regard, previous research points towards the crucial role experimental pond studies may play for providing unique insights into fish-angler interactions. For example, by releasing fish artificially selected for either a low or high vulnerability to angling in a series of replicate ponds, interdependencies among angling vulnerability and other traits (e.g. metabolism, behaviour, growth-rate) have been uncovered [105, 106]. Moreover, experimental amplification of heritable intraspecific variation in trait vulnerability to angling combined with release in controlled ponds, experimental fishing and subsequent genetic assignments of parent-offspring relations showed that anglers may selectively target fish individuals with the highest reproductive potential [107], highlighting that fish is harvested in a non-random fashion [108]. Hence, we believe that using whole-pond acoustic telemetry studies will continue to further our understanding of fish-angler interactions and especially on the sub-lethal, trait-mediated effects that may be associated with contemporary angling practices.

Many fish individuals caught by anglers are released back to the water either because the angler have to comply with fishing regulations, such as length regulations, bag limits or closed seasons or because the angler prefer to release the fish voluntarily, and this procedure is often referred to as catch-and-release (C&R) angling [109, 110]. Here, an implicit assumption is that the effects on fish individuals are transitory and do not reduce growth/survival. This can be tested in experimental ponds by stocking with controlled fish assemblages (preferably captured by active gear, such as electrofishing, to maximize standing phenotypic variation) and monitor changes in movement patterns before and after exposure to angling. Studies can even be extended to include how angling affects key internal state variables, such as immune function, which is fundamental for disease susceptibility, as well as physiological and behavioural stress indicators via implanted bio-loggers (e.g. heart-rate tags). By extending the sampling period well beyond the impact phase, and through putatively critical time windows (e.g. spawning periods) we can also monitor recovery trends in these traits [110] and directly assess the potential for demographic consequences of C&R angling (e.g. by retrieval and direct comparisons of produced offspring fished/unfished ponds).

Furthermore, tracking changes in fish social networks in response to recreational angling is a key priority since non-random associations between individuals are primary drivers of many ecological and evolutionary processes and play an important role in understanding the resilience of biological systems [111, 112]. A controlled fish community, in which all individuals are tagged and surveyed by whole-pond acoustic telemetry, allows us to address outstanding questions requiring considerable data on the repeated interactions or associations of multiple individuals. For example, how does individuals change the connectivity and strength of their communication as a function of angling? How does fisheries-induced selection operate on different social/asocial behaviours, i.e. are individuals captured by recreational anglers especially highly connected, dominant or perform important social functions? Moreover, if stress cascades from angled to non-angled individuals, for example via social transmission [113], the total impact of recreational fishing on fitness may be far greater than previously anticipated. Or, alternatively, can grouping with conspecifics act to mitigate C&R-induced stress responses (“social buffering” [114] associated with catch and release events?

## Limitations and challenges

Pond facilities offer a solid experimental platforms where mechanistic studies can be performed on a scale that is relevant to natural systems; sampling of small ponds in the region show that ponds of similar sizes as the iPonds holds persistent, natural fish assemblages [20]. However, their temporal and spatial scale is still a limitation since not all processes and mechanisms could be studied at a small, pond scale. For example, the experimental ponds are shallow and do not show seasonal stratification patterns and thus we cannot make inferences for fish movement patterns in deeper lakes with thermal stratification and anoxic waters during parts of the year. Further, small ponds are obviously not a useful venue to target questions on large-scale movement patterns, such as spawning migrations and seasonal migration driven by resource availability/predation threat trade-offs [115–117]. Many studies on animal movement using acoustic tags in freshwater and marine environments have been justified by the need to increase our knowledge in order to make correct decisions in conservation management issues, i.e. where to place protected areas and how to increase habitat connectivity in migrating species [13, 29] and these questions also need to be answered in larger systems. One of the advantages of the experimental ponds is that we can have full control over all fish individuals by stocking previously empty ponds (see above), but this also means that fish assemblages to some extent still are simplified as compared to natural fish communities, for example with regard to species and size composition and population dynamics over longer time periods. Quantifying movement patterns on a still rather limited time frame, compared to the generation time of many fish species, makes it challenging to link movement patterns to ecological and evolutionary time scales and to study e.g. lifetime fitness parameters or the evolution of traits that incur benefits/costs for movement (e.g. [88]).

With regards to fish stocking, some experiments, particularly on adult fish, may require that focal fish are captured in systems in which they have experienced similar abiotic (e.g. water chemistry, temperature) and biotic (e.g. types and amounts of predators and competitors) conditions to decrease acclimation times and increase the likelihood that researchers capture ecologically relevant behaviours. Alternatively, stocking fish originating from different environmental backgrounds can offer opportunities to address questions regarding behavioural establishment in novel environments (e.g. [118]). With regards to fish stocking, we also see ample opportunities to develop or take advantage of already established fish stocking facilities, which will allow researchers to study fish individuals reared in highly controlled background environments (e.g. food availability, predation risk) or conduct selective breeding experiments (e.g. artificial selection) to experimentally produce phenotypes relevant for examining genetic variances and covariances underlaying complex movement traits of ecological significance.

It has earlier been suggested that extensive coverage of submerged macrophytes may affect signal detection range or that ice cover during winter may reflect or distort acoustic signals and acoustic noise during spring ice break-up may decrease detection range [119]. We also need to consider ethical issues when we design our experiments and take great care so that potential negative effects of implanting acoustic tags are kept at minimum and sample sizes are kept as low as possible, although the use of continuous acoustic telemetry can gather highly quantitative data, which should act to minimize exhaustive oversampling.

Although experimental pond systems enable remote sensing of fish behaviour at very high resolution, both via positional telemetry and high frequency biologging, there is still a large repertoire of important behaviours expressed by fish that currently are difficult to capture reliably via remote sensing, such as aggressiveness, various display behaviours, detailed spawning behaviour and selective foraging. Here, researchers are still dependent on observational studies in e.g. aquaria, which severely limit the ability to make relevant ecological connections to natural systems. Artificial intelligence is increasingly being used on high-resolution accelerometer and tilt data [120] as well as tracking data [121] to identify particular behaviours and such approaches may in the future enable more subtle behaviours to be detected in ponds.

A challenge that remains also in the near future is the handling and analysis of the huge data amounts that are produced in movement studies where the position of a large number of individual fish are collected at a high temporal resolution using an array of many signal receivers and over extended periods of time. The analysis of such datasets of course meets large challenges and requires advanced analytical and statistical methods. However, we now see very rapid developments of for example machine learning methods that should provide an important tool for analysing the large data sets generated by acoustic telemetry studies (e.g. [7]).

Finally, with regards to access to ponds we realize that we have been very fortunate having had a long-term collaboration with South Sweden Water Supply AB (Sydvatten AB) that allowed us to expand our studies to aquatic movement ecology by implementing acoustic telemetry systems in a series of already available and maintained infiltration ponds. We acknowledge that such an opportunity may not be available to all and that identifying already existing ponds that meet key criteria with regards to experimental control (e.g. appropriate size and possibilities to regulate water-level) could provide a major challenge. Constructing new ponds will of course allow researchers to tailor ponds specifically to research needs, but this is in most cases prohibitively costly to be included in research grant budgets. We can here only encourage researchers interested in utilizing ponds for studying movement ecology to explore opportunities for collaborations with other research institutions, angling associations, fish farmers and water works facilities that may maintain ponds suitable for their research goals.

## Concluding remarks

Movement ecology studies have proliferated in the past decade, largely because new paradigms and technical innovations have pervaded the field, providing increasingly powerful means to deliver fine-scale movement data, attracting renewed interest. Specifically, in the aquatic environment, tracking with acoustic telemetry now provides integral spatiotemporal information regarding individual movement in the wild. We have tried to highlight that this technology also holds great promise for experimental studies, and thus for our ability to truly establish cause-and-effect relationships in animal movement studies, and that ponds with their well-defined borders are particularly suited as empirical substrates to achieve this. We have shared our vision of how experimental movement ecology will develop in the future and provided explicit examples on how replicated ponds equipped with modern aquatic telemetry systems offer opportunities for experimentation, allowing researchers to control and manipulate factors such as temperature, light, habitat structure, food availability, and predator presence/absence. We believe that experiments conducted in ponds, designed to reveal the how, when, where, why, and which animals move, can provide valuable insights into the underlying mechanisms and paradigms that govern movement phenomena. These insights may extend well beyond ponds and swimming animals to other realms, such as terrestrial environments and animals that move on foot or by flight. Moreover, many spectacular animal movement phenomena have either disappeared or are in steep decline due to the global biodiversity crisis, meaning that unravelling the conundrum of movement ecology is of profound applied importance. We are confident that animal movement studies in ponds can help to better understand how moving animals are linked to population-level processes and provide us with a great lens through which to study the impact of environmental change. Given the continued reliance of movement ecology on experimental approaches and hypothesis testing, ponds are expected to remain invaluable experimental arenas. We eagerly anticipate the discoveries that will be made in the coming years.

## Data Availability

Not applicaple.
